# Women's Experiences with a Perinatal Peer Support Specialist Program

**DOI:** 10.1097/NMC.0000000000001154

**Published:** 2025-12-03

**Authors:** Kalyn M. Renbarger, Jean Marie Place, Corie Hess, Lina Burkhart

**Affiliations:** **Kalyn M. Renbarger** is an Assistant Professor of Nursing at Purdue University, West Lafayette, IN. Dr. Renbarger can be reached at kmrenbar@purdue.edu; **Jean Marie Place** is an Associate Professor of Public Health at Ball State University, Muncie, IN. Dr. Place can be reached at jsplace@bsu.edu; **Corie Hess** is a Psychologist at Open Door Health Services, Muncie, IN. Dr. Hess can be reached at cllhess@bsu.edu; **Lina Burkhart** is an Assistant Clinical Professor in the Department of Counseling Psychology, Social Psychology, and Counseling at Ball State University, Muncie, IN. Dr. Burkhart can be reached at llburkhart@bsu.edu

**Keywords:** COVID-19, Mental health, Parenting, Pregnancy, Women

## Abstract

**Purpose:**

The purpose of this study is to describe the experiences of women with symptoms of perinatal mental health disorders who recently gave birth and participated in a virtual peer support specialist program for maternal mental health during the COVID-19 pandemic.

**Study Design and Methods:**

A qualitative descriptive design was used to describe the experiences of six women with symptoms of perinatal mental health disorders who participated in a peer support specialist program. Participants were recruited from online Indiana-based Facebook parenting groups targeted to women with young children. Semi-structured interviews were conducted, and data were analyzed using a basic inductive content analysis.

**Results:**

We identified three main themes: 1) Seeking help for mental health symptoms, 2) Receiving unbiased peer support, and 3) Improving mental health symptoms.

**Clinical Implications:**

Implications for nursing practice include developing strategies to increase peer networks for women in the perinatal period, increasing the screening of women for symptoms of perinatal mental health disorders, and conducting more research on the efficacy of a peer support specialist programs for improving maternal mental health.

## Abstract 2

In this study, the experiences of women with symptoms of depression and anxiety in the perinatal period who participated in a virtual peer support specialist program were evaluated. Analyis of interviews with program participants allowed identification of three themes: seeking help for mental health symptoms, receiving unbiased peer support, and improving mental health symptoms. Virtual peer support programs may be helpful for women with perinatal anxiety and depression.

**Figure:**
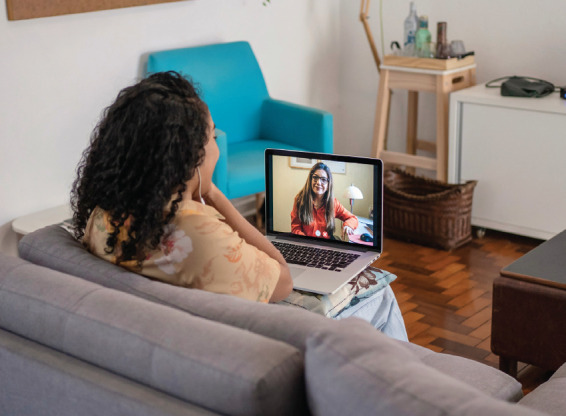


Perinatal mental health disorders are a critical public health concern in the United States. Perinatal mental health disorders include symptoms of depression and anxiety and are commonly experienced by women in the perinatal period in the United States. The Diagnostic and Statistical Manual of Mental Disorders (DSM-5) defines perinatal depression as a major depressive episode that begins during pregnancy or within 4 weeks after birth (American Psychological Association [APA], 2013). Symptoms of perinatal depression include feelings of sadness, an “empty” mood, and hopelessness; insomnia or hypersomnia; feelings of worthlessness; and irritability (APA, 2013). There is no formal DSM-V label for perinatal anxiety. However, the DSM-V recognizes symptoms of general anxiety disorder which include excessive worry, restlessness, fatigue, irritability, and sleep disturbance (APA, 2013). Rates of perinatal mental health disorders are on the rise in the United States (US Department of Health and Human Services, n.d.). Weiss et al. (2022) found that between 2017 and 2020, the rate of delivery stays involving mental health disorders increased by 52%. Likewise, suicide was associated with 8.4% of pregnancy-related deaths between 2017 and 2019 (Trost et al., 2022) which was increased from another report of 5.7% in 2017 (White et al., 2023). With perinatal mental health disorders, there are increased rates of preterm births (9.7 vs. 6.7), lengths of stays (3.1 days vs. 2.6 days), and birth-related costs ($5,200 vs. $4,400) among women with perinatal mental health disorders compared to other women without perinatal mental health disorders (McKee et al., 2020).

Obstetric risk factors such as severe maternal morbidity have been linked to perinatal mental health disorders (McKee et al., 2020; Weiss et al., 2022). Symptoms of perinatal mental health disorders have been associated with decreased breastfeeding, which can negatively affect maternal–infant health (Sanni et al., 2024; Toledo et al., 2022). Likewise, postpartum depression has been linked to delays in child development including language skills, personal–social skills, and fine motor and adaptive skills (Lubotzky-Gete et al., 2021; Zhu et al., 2024).

Despite the mental health challenges of the perinatal period, there are promising interventions that may help alleviate symptoms of perinatal mental health disorders. Women in the perinatal period have reported benefits from peer support that include increased maternal self-confidence, self-esteem, and hope for recovery from mental health symptoms (Carter et al., 2020; Kurniawati et al., 2019). A promising peer intervention for mental health is the use of a peer support specialist. Peer support specialists provide recovery and wellness support services provided by an individual with a lived experience of recovery from a mental health condition (Fortuna et al., 2022). Peer support specialists have been used to address mental health concerns for presenting concerns like substance use, youth depression and anxiety, and post-traumatic stress disorder (Scannell, 2022; Simmons et al., 2023; Smith et al., 2020). Research has suggested positive outcomes for those who use peer support services, as well as for those who function in the peer support specialist role (Carter et al., 2020; Scannell, 2022).

The COVID-19 pandemic additionally challenged the mental health of women in the perinatal period (Hessami et al., 2022; Penna et al., 2023) as they were often fearful of COVID-19 exposure for themselves and their fetus or newborn infants (Goyal & Selix, 2021; Penna et al., 2023). Due to efforts to decrease the spread of COVID-19 (i.e., quarantine and social distancing), women in the perinatal period often received limited support during the pandemic (Chivers et al., 2020; Goyal & Selix, 2021; Penna et al., 2023) and more health care services moved online to telehealth options (Hawkins, 2023; Molfenter et al., 2021). During the COVID-19 pandemic, the management of maternal mental health was a challenge due to an intensifying national shortage of mental health providers. Due to restrictions placed to prevent the spread of COVID-19, telehealth was often used to deliver health care and mental health services (Hawkins, 2023; Molfenter et al., 2021).

Little is known about how women with symptoms of perinatal mental health disorders experienced mental health interventions during the COVID-19 pandemic. This study sought to answer the question: How do women with symptoms of perinatal mental health disorders experience a peer support specialist program for maternal mental health? More information is needed on peer support specialist interventions specifically to enhance program components and tailor strategies to promote maternal mental health more broadly. The purpose of this article is to describe the experiences of women with symptoms of perinatal mental health disorders who participated in a pilot virtual peer support specialist program during the COVID-19 pandemic.

## Methods

### Design

This study was conducted using a qualitative descriptive design, which is frequently used in nursing research to explain an event with low interpretations from the researcher (Bradshaw et al., 2017; Sandelowski, 2000). This approach is also appropriate to use for straightforward descriptions of experiences when little is known about the phenomenon being studies (Sandelowski, 2000). Individual, semi-structured interviews are the most common data collection method (Kim et al., 2017). Qualitative description was deemed to be the most appropriate study design because the goal of this study was to provide straightforward descriptions of experiences in a virtual peer support specialist program among women with symptoms of perinatal mental health disorders during the COVID-19 pandemic.

The virtual peer support specialist program used certified peer support specialists who 1) were at least 18 years old; 2) had experience with symptoms of depression and anxiety in the perinatal period; 3) were assessed for recovery and deemed qualified to serve as a peer-support specialist by a licensed psychologist; and 4) reported living in the state of Indiana. Each of the peer support specialists were certified for mental health through the Mental Health America of Northeast Indiana (n.d.) and received a 5-day, formal certificate training prior to working with participants. Peer support specialists also received an additional 2-day virtual certificate training for maternal mental health through Postpartum Support International (PSI) titled “Perinatal Mood Disorders: Components of Care.” The maternal mental health training is an evidence-based curriculum designed for nurses, physicians, social workers, mental health providers, childbirth professionals, social support providers, or anyone interested in learning skills and knowledge for assessment and treatment of perinatal mental health disorders (PSI, n.d.-a). The peer support specialists met regularly with the research team to discuss concerns or troubleshoot challenges. Each peer support specialist was paired with a licensed psychologist from the research team to be able to consult about mental health concerns of participants, if needed. Peer support specialists received a $200 gift card for their participation in the program.

The virtual peer support specialist program was announced via Indiana-based Facebook parenting groups targeted to women with young children. Women with perinatal mental health disorders were recruited to participate in the virtual peer support specialist program if they 1) were 18 years of age or older; and 2) self-reported current symptoms of perinatal mental health disorders during their most recent pregnancy or within 1 year of childbirth. Women were able to participate if they experienced symptoms during pregnancy and/or within 1 year postpartum but were outside the perinatal period at the time of the study. There is growing evidence to support that perinatal mental health disorders and their effects can extend beyond the perinatal period through maternal genetic pathways (Nicoloro-SantaBarbara et al., 2022).

Women with perinatal mental health disorders indicated their interest by completing a brief online survey and in which they provided their phone number and/or email. Interested women with perinatal mental health disorders were contacted and received information about the program. Participants (i.e., women with perinatal mental health disorders) were paired with a peer support specialist based on similarities in backgrounds and the availability of the peer support specialist. All sessions between the participants and their peer support specialist were completed virtually through a series of between 6 and 8 Zoom or phone calls. Each individual session lasted between 30 and 60 minutes. The peer-to-peer mentoring sessions consisted of the peer support specialists providing emotional support and encouragement, sharing of lived experiences, providing information about community resources, and affirming the stories of women with symptoms of perinatal mental health disorders. The peer-to-peer sessions aimed to normalize conversations around maternal mental health by promoting a supportive environment between two individuals with shared lived experience. The peer support specialist program was conducted between January 2022 and April 2023 with each individual peer-to-peer session lasting between 6 and 8 weeks during that time.

### Procedures

The study was approved by the institutional review board of the researchers' institution. After completion of the peer support specialist program, participants of this study (i.e., women with perinatal mental health disorders) were emailed an informed consent form which explained the study in detail. The researcher set up a time to discuss the study over phone, answer questions, and obtained recorded verbal consent from the participants before beginning the interview. Questions in the interview guide included, “How did it feel to have a peer support specialist talk with you?” “Describe your mental health symptoms before starting the peer support program. Did any changes occur after completing the peer support program?” The interviews were audio recorded and lasted approximately 30 minutes. Participants were given a $50 gift card after completion of the interview.

### Analysis

The participants' audio recordings were transcribed through an IRB–approved professional transcriptionist. First, the research team read the transcripts. The data analysis approach included a basic content analysis outlined by Kyngäs (2020). The three steps to a basic content analysis emphasized by Kyngäs (2020) were used to guide the analytic process. The three steps included: 1) data reduction, 2) data grouping, and 3) the formation of concepts that can answer the research question. First, each text unit related to the experiences of women with symptoms of perinatal mental health disorders in a peer support specialist program was highlighted and assigned a code by the PI. Codes were verified by the second author (JMP). The codes were then grouped to form themes. An audit trail of the research process was kept by the PI to enhance dependability of the study. This study also provides a detailed description of the study sample to enhance the transferability of the findings to other contexts.

## Results

Ten women with symptoms of perinatal mental health disorders consented to participate in the peer support specialist program. Three participants could not be reached to begin the peer support specialist program and therefore seven participants completed the peer support specialist program from January 2022 to April 2023. Out of the seven participants, six completed the phone interview at the end of the program. One participant was pregnant at the time of the study. Four participants were within the postpartum period. One participant reported symptoms which began during pregnancy extended beyond the postpartum period and was parenting a 3-year-old child. Demographic characteristics can be found in Table 1.

**TABLE 1.** Participant Characteristics

Characteristic	Results	Percentage (%)	Mean (*N*=6)
**Age in years**			26.66 years
24-39 years	6	100	
**Number of Children**			1.83 children
1 child	3	50	
2 children	2	33.3	
3 children	0	0	
4 children	1	16.7	
**Self-Identified Race/Ethnicity**			
Biracial/Mixed Race	1	16.7	
Hispanic/Latina	1	16.7	
White/non-Hispanic	4	66.7	
**Gender**			
Female	6	100	
**Marital Status**			
Married	2	33.3	
Single	2	33.3	
Engaged	1	16.7	
Separated	1	16.7	

Three themes were identified from the data and included: 1) Seeking help for mental health symptoms, 2) Receiving unbiased peer support, and 3) Improving mental health symptoms. Themes and exemplar quotes are displayed in Table 2.

**TABLE 2.** Themes and Exemplars

Themes	Exemplars
**Seeking help for mental health symptoms**	*It [COVID-19 pandemic] took a really, really bad toll on me mentally because I didn't have family around.*
	*A lot of anxiety was involved with this pregnancy.*
	*I had anxiety and depression.*
**Receiving unbiased peer support**	*She [peer support specialist] talked to me when I had trouble. She was always there when I needed her, and she told me some resources.*
	*She [peer support specialist] was looking for things to help me out.*
	*She didn't have a biased opinion at all because she didn't know me.*
**Improving mental health symptoms**	*[Meeting with the peer support specialist] gave me something to look forward to.*
	*[My mental health] was a little better, knowing that I have somebody I could talk to if I needed her [peer support specialist].*
	Then it's [mental health] gotten a little bit better because things have normalized.

### Seeking Help for Mental Health Symptoms

Participants described symptoms of depression and anxiety as motivation to seek the peer support specialist program and to continue visiting with their peer support specialists. Participants described situations that worsened their mental health symptoms and served as motivation to seek help from a peer support specialist. One participant (p2) recalled the grief of a past pregnancy loss as she was coping with a new pregnancy; *So, there's a lot of anxiety and kind of going through some of the grief of like, you know, grieving the past life with this new life*. A participant (p3) referred to her mental health as being *in the trash can* and stated she was 3 hours away from anyone who *cares anything about me* which served as a motivator to seek out the peer support specialist program.

Participants indicated that the COVID-19 pandemic was mentally challenging for them and worsened their symptoms of depression and anxiety. Participants reported feeling isolated and experiencing changes that were out of their control during the pandemic. Participants also reported that their usual sources of social support (i.e., classes for women) were closed during COVID-19. Participants discussed decreased contact with family and friends due to fears related to the spread of COVID-19. One participant (p1) stated, *I'm going crazy over here, and I have to take care of her [child] and nobody else can*. She indicated that she did not have medical insurance and could not afford to pay for counseling services out of pocket, so the peer support specialist program offered her an alternative. She stated, *Oh, this would be great, just talking to somebody*.

### Receiving Unbiased Peer Support

Participants described how receiving unbiased support from their peer support specialist was beneficial and encouraging to them during a stressful time in their lives. Participants described various ways in which they established a personal connection to their peer support specialist. For instance, one participant (p1) recalled how her peer support specialist was able to personally relate to her situation of having a single child. Participants believed the resources their peer support specialists provided were useful and helpful. One participant (p5) stated, *I was able to actually use some of the tips that she gave me*. She went on to suggest that her peer support specialist would then revisit the tips and ideas at the next visit which helped her to feel supported. Participants enjoyed that their peer support specialists did not know them and did not hold biased opinions of them. One participant (p2) indicated, *since she [peer support specialist] doesn't know my family, she is able to offer maybe more realistic help. That [help] was not emotionally based. I was having some mental health issues, and she came at it from a good angle of someone who understands and was able to offer her advice.*

Another participant (p1) recalled a similar experience and stated, *It's nice to just have somebody that doesn't know me, talk to me about how everything is going. That definitely was helpful just to have somebody*. The participant (p1) appreciated that her peer support specialist did not judge her parenting style. Participants enjoyed having the peer support specialists with shared lived experiences checking on them, motivating them, and offering them emotional support.

### Improving Mental Health Symptoms

Participants recalled having improved symptoms of depression and/or anxiety at the completion of the program. Several participants stated that they looked forward to each session with their peer support specialists and having someone they could reach out to when needed which decreased their levels of anxiety and depression. Participants believed that having someone to talk to was significant in improving their mental health symptoms. Participants sometimes felt isolated at home without *adult interaction* in the house and the peer support specialists served as an outlet where they could freely express their feelings and concerns. One participant (p4) stated, *It actually helped because I had someone I actually like to talk to.* Another participant (p5) recalled, *it's just really nice to talk to somebody, so I guess it did make my mental health better*. Another participant (p1) recalled her experiences with her peer support specialist and stated, *I think just things [mental health] got a little bit easier…. but [I] haven't been doing too bad I would say*.

Participants appreciated receiving affirmation of their feelings by their peer support specialist which reduced their stress and improved their mental health. One participant (p1) stated, *I'm not on any meds for my anxiety, but it was nice to just talk to someone and see if I was going crazy or not. If it [mental health] was bad for that week, I could reach out be like [to the peer support specialist], Hey, this is what's going on.*

## Discussion

Women with symptoms of perinatal mental health disorders in this study found their peer support specialists to be a source of emotional support, encouragement, and hope, which they perceived had a positive effect on their mental health. These findings are consistent with other studies where women have improved their mental health by developing deep connections with peers who have a shared experience (Gruß et al., 2021; Paterno et al., 2018). There is a growing body of literature that suggests the effectiveness of peer support interventions for maternal mental health. In a systematic review and meta-analysis, Huang et al. (2020) found peer support intervention to be well received by women experiencing perinatal depression and improved levels of depression. They suggested that peer support interventions may be effective in preventing or reducing the harm from perinatal depression (Huang et al., 2020). Having access to peer support specialists has been associated with increased perceptions of support, decreased substance use, and increased engagement in medical services for women in the perinatal period (Cos et al., 2020; Olding et al., 2022).

Participants perceived the COVID-19 pandemic as contributing to an increase in symptoms of perinatal mental health disorders which served as a catalyst to seeking peer support. This is consistent with prior literature that have suggested the negative impact the COVID-19 pandemic had on maternal mental health. Women experienced significant stress symptoms (40%), social isolation and loneliness (62%), feelings of irritability and feeling down (>50%), and worry (>70%; Wall & Dempsey, 2023). Likewise in a systematic review and meta-analysis, Hessami et al. (2022) provided evidence that the COVID-19 pandemic increased the risk of anxiety and depression among women in the perinatal period.

Several limitations should be considered in this study. The sample size is small and from one geographical location; however, this is generally suitable for qualitative studies. No comparisons could be made among the sample regarding cultural, racial/ethnic, gender, and other factors. More research is needed to understand participants' motivation or lack of motivation to participate in a peer support specialist program. Selection bias should also be considered a limitation. It is possible that participants who chose to participate in the peer support specialist program have differing views than other women with perinatal mental health disorders who did not choose to participate.

## Clinical Implications

Nurses should acknowledge the need to offer more services, screening, and support for women experiencing perinatal mental health disorders. The American College of Obstetricians and Gynecologists (2023) recommends that all maternity care health care professionals (i.e., nurses, midwives, physicians) complete an assessment for depression and anxiety at least once in the perinatal period using a validated instrument. Recognizing that maternal mental health has been negatively affected by the pandemic, extended screenings may be necessary that can extend beyond pregnancy and the immediate postpartum period. The American Academy of Pediatrics recommends expanding screening for perinatal mental health disorders through the infant's 1-, 2-, 4-, and 6-month well-child visits (Lamere & Golova, 2022). Extending screening for perinatal mental health disorders is essential to early diagnosis and comprehensive support to women who have experienced unique challenges brought on by the COVID-19 pandemic (Hanna et al., 2004). The United States Preventive Services Task Force (USPSTF) recommends that women who are pregnant or in the postpartum period and at risk for depression be referred to counseling interventions based on the results of a systematic review of 50 studies (O'Connor et al., 2019).

Nurses should be aware of peer support services offered by PSI (n.d.-b) along with community-based peer support programs and refer women in the perinatal period to this service. Postpartum Support International (PSI, n.d.-b) offers a peer mentor program where individuals experiencing perinatal mental health disorders can relate to another individual with the lived experience and recovery from perinatal mental health disorders. The program offers encouragement from someone who has experienced a perinatal mental health disorder by establishing a one-to-one connection with an individual who is struggling. Peer support specialist interventions can supplement existing maternal care settings where factors such as a lack of time or privacy can be problematic. Peer-led interventions have potential to break stigma surrounding perinatal mental health disorders, promote resilience, and foster a sense of connection to individuals who are experiencing symptoms of perinatal mental health disorders (Sun et al., 2022).

There is a need for an increase in peer support specialist training programs that are accessible and affordable. More rigorous studies such as randomized controlled trials are needed to assess the impact of a peer support specialist intervention on perinatal mental health disorders for women in the perinatal period. Future research should compare outcomes of in-person versus virtual models of peer support specialist programs. Research needs to be conducted to evaluate feasibility and sustainability to integrate the intervention and serve as a complementary service to traditional health care treatment options.

### Acknowledgment

This project was supported by the Indiana Clinical and Translational Sciences Institute, funded in part by grant #UL1TR002529 from the National Institutes of Health, National Center for Advancing Translational Sciences.

## CLINICAL IMPLICATIONS

- Nurses can assist women with perinatal mental health disorders in identifying and establishing peer networks.
- Nurses should increase the screening of women for symptoms of perinatal mental health disorders.
- Nurses can incorporate extended screening women for symptoms of perinatal mental health disorders into well-child visits after childbirth.
- Nurses can refer women to peer support specialist programs.
- Nurses can conduct more research on the use of peer support specialist program for improving maternal mental health.
